# A New Quantitative Classification of the Extrahepatic Biliary Tract Related to Cystic Duct Implantation

**DOI:** 10.1007/s11605-020-04852-8

**Published:** 2020-12-02

**Authors:** Matteo Renzulli, Stefano Brocchi, Giovanni Marasco, Daniele Spinelli, Caterina Balacchi, Massimo Barakat, Irene Pettinari, Rita Golfieri

**Affiliations:** 1 Department of Radiology, IRCCS Azienda Ospedaliero-Universitaria di Bologna, Via Albertoni 15, Bologna, Italy; 2 Department of Medical and Surgical Sciences, S. Orsola Hospital, University of Bologna, Bologna, Italy

**Keywords:** Anatomy, Biliary tract, Cystic duct, Cholangiopancreatography, Magnetic resonance, Lithiasis

## Abstract

**Background:**

Knowledge regarding biliary anatomy and its variations, including the cystic duct (CD), is important in the pre-surgical setting and for predicting biliary diseases. However, no large series has focused on CD evaluation using a quantitative analysis. The primary aim of this prospective study was to create a ‘taxonomic’ classification of CD anatomy in a large cohort of subjects who underwent magnetic resonance cholangiopancreatography (MRCP). The secondary aim was to evaluate the correlations between extrahepatic bile duct (EHBD) variants and biliary diseases.

**Methods:**

We enrolled patients who underwent MRCP for different clinical indications from January 2017 to May 2019. Demographical, anatomical and clinical data were evaluated using statistical analyses, as appropriate. The anatomical assessment of EHBD was performed using the standard classification for CD in low, medium, and high insertions, and the lengths of CD to the duodenal papilla (DP), and EHBD was determined to conduct a new quantitative analysis.

**Results:**

The final study population comprised 1004 subjects. A new classification for EHBD as per the percentile distribution of the ratio CDDP/EHBD was designed, and the following categories were obtained: type 1 (below the 25th percentile) for CDDP/EHBD ratio ≤ 50%; type 2 (25th to 75th percentile) for CDDP/EHBD ratio 51–75% and type 3 (above the 75th percentiles) for CDDP/EHBD ratio > 75%. Type 1 of the new classification of CD implantation was significantly superior in terms of the detection of low, medial and intra-pancreatic CD that was significantly correlated with a high risk of choledochal lithiasis in comparison with the standard classification (*P* < 0.001).

**Conclusions:**

The new classification of CD implantation enables identification of the vast majority of intra-pancreatic CDs that are correlated with a high risk of choledochal lithiasis in a single category (type 1) that is easy to identify using imaging.

**Supplementary Information:**

The online version contains supplementary material available at 10.1007/s11605-020-04852-8.

## Introduction

Considering the arrangement of the segmental bile ducts, the ‘classical’ anatomy of the intra-hepatic biliary tree includes the right posterior duct (RPD) draining the liver segments VI and VII and the right anterior duct (RAD) draining the liver segments V and VIII to join the right hepatic duct (RHD). Biliary ducts of segments II, III and IV unite to form the left hepatic duct (LHD). The RHD and LHD join in the common hepatic duct (CHD) at the hepatic hilus.^1^,^2^ After the confluence of the cystic duct (CD), the CHD forms the common bile duct (CBD) that finally drains the bile into the duodenum through the papilla of Vater.^1^,^3^

This normal biliary anatomy of the intra-hepatic ducts is present in 58–64.5% of the population.^2^,^4^ Many anatomic variations of the intra-hepatic ducts have been described in the literature, particularly RPD that is more often described in its different insertions.^1^,^4^,^5^ In addition, with respect to the extrahepatic biliary ducts (EHBDs), the described normal anatomy is present in only approximately 53% of the population.^6^ Although the anatomical variations of the EHBD have also been investigated, the anomalous pancreatic–biliary junctions and choledochal cysts have always been the main focus of most published series.^7^,^8^

The CD, which measures 2–4 cm in length, connects the gallbladder to the CHD and typically inserts into the middle third of the EHBD usually on the right side.^1^,^2^,^9^ The anatomical variations of CD insertion are historically less investigated than those of other biliary tracts^7^,^8^; they are reported in 25% of individuals and concern the radial attachment of CD into the wall of EHBD in the lateral, posterior or medial side^4^,^9^,^10^ or the implantation in the lower or higher third of the EHBD or into the RHD, resulting in abnormal length or shortness of CD itself. Another abnormal low CD insertion involves the intra-pancreatic junction and is characterised by the CD joint with the intra-pancreatic portion of the EHBD.^1^,^3^

Biliary tract diseases represent a very common medical problem and often require emergent interventions. For example, cholelithiasis affects approximately 10% of the adult population, with the prevalence increasing with age.^11^ Approximately 35% of this population could be affected by complications and symptoms that could require cholecystectomy.^11^ Furthermore, the lack of knowledge in this surgical area could create many complications for patients, ranging from infections to definitive or even lethal injuries.^4^,^8^,^11^,^12^ Moreover, many other diseases could affect the biliary tree, representing very common causes of hospitalisation and surgical treatments.^9^,^13^ Biliary variants, including those of the CD, can be the direct cause of different diseases. Low insertion of the CD has a stronger association with CBD stone formation, CBD dilatation and positive bacterial culture from the bile than CD with a normal joint with the EHBD.^10^,^14^ Therefore, it is crucial to acquire appropriate knowledge about normal and variant anatomies of intra-hepatic and extrahepatic biliary systems.^4^

Magnetic resonance cholangiopancreatography (MRCP) is the most accurate imaging modality for assessing the intra-hepatic and extrahepatic bile tracts and the CD owing to its multiple abilities^15^–^17^; it is the preferred non-invasive technique for evaluating the biliary tract if immediate therapy for a known problem is not the primary aim.^18^

Although establishing the correct diagnosis of CD variants is essential for assessing subjects at higher risks of both spontaneous and surgical bile duct injury,^1^,^10^,^14^ to the best of our knowledge, no large series has focused on the evaluation of CD variants using MRCP.^9^,^19^ Moreover, the vast majority of the published studies that have been conducted with the aim of evaluating CD implantations have only performed qualitative analysis.^1^,^2^,^9^ In fact, all the evaluations concerning the site of the CD joint into the EHBD start from a descriptive point of view, reporting a generic (not quantitative) insertion into the ‘proximal’, ‘medium’ or ‘distal’ third of the EHBD.

The primary aim of this study was to create a ‘taxonomic’ classification of the CD anatomy in a large prospectively collected cohort of subjects undergoing MRCP, reporting all the anatomical variants of intra-hepatic and EHBDs. The secondary aim was to evaluate the correlations between the anatomical variations in the EHBDs and the diagnosis of biliary tree diseases.

## Materials and Methods

The local institutional review board approved this prospective study, and written informed consent was obtained from all the patients. This study was conducted according to the Declaration of Helsinki for clinical studies.

### Patients and Imaging Technique

All the patients who underwent MRCP for multiple indications from January 2017 to May 2019 at our institution were enrolled. The following demographical data of the patient population were collected in a dedicated database: age, sex, previous cholecystectomy and any indications for MRCP other than previous diagnosis of biliary tree disease (follow-up MRCP). All the MRCP examinations were performed as per a standardised protocol that has been previously described in detail,^15^,^16^ using the same single 1.5 T MRI superconductive scanner (HDX-t Signa; General Electric®, Milwaukee, WI, USA). The examinations were evaluated by two radiologists—one (MR) with more than 15 years of experience in hepato–bilio–pancreatic disease and the other (SB) with 8 years of experience in the same radiological field. All MRCPs were evaluated on a PACS workstation (Carestream Vue Solutions, version 11.4.1.1011, Carestream Health Italy).

### Image Analysis

The following data were collected for each MRCP examination: (1) type of intra-hepatic biliary anatomy; (2) length between CD insertion and the duodenal papilla (CDDP) and the length of the EHBD (CHD plus CBD); (3) circumferential (radial) insertion of the CD into the EHBD; (4) presence or absence of intra-pancreatic CD; (5) presence or absence of lithiasis in the gallbladder or the EHBD and (6) the final diagnosis of benign or malignant diseases.

The variants of intra-hepatic biliary tract anatomy were recorded as per the following previously published classification^4^: (I) type 1, conventional biliary anatomy, defined as the formation of the RHD by the anterior and posterior branches and convergence of both the RHD and the LHD into the CHD; (II) type 2, a trifurcation pattern with a common confluence of the RPD, RAD and LHD; (III) type 3a, characterised by the RPD draining into the LHD; (IV) type 3b, wherein the RPD drains into the CHD and (V) other anomalies of the intra-hepatic bile ducts. It was decided to singularly record the first four types of variants (types 1, 2, 3a and 3b) because together they accounted for 98.5% of all the possible variations.^3^

For EHBD assessment, it was decided to overcome the qualitative approach, such as the classification of CD insertion in low, medium and high, performing a detailed quantitative analysis. Therefore, the following measurements were performed: (1) the CDDP length, expressed in millimetres (mm) and (2) the EHBD length (CHD plus CBD), calculated as the distance between DP and the confluence of the RHD and LHD, expressed in mm. These two measurements allowed us to apply the ‘conventional’ classification terminology of CD joint as low, middle and high implants with a quantitative approach and, moreover, to experience new quantitative analysis as well (see the “Statistical Analysis” section).

The radial CD insertion into the EHBD was recorded as lateral, posterior or medial.

The presence or absence of CD with an intra-pancreatic insertion was also recorded. In the absence of an unequivocal and widely accepted definition of intra-pancreatic CD, it was decided to define the intra-pancreatic CD as that when the CD joined the EHBD in its tract contained into the pancreatic parenchyma, irrespective of the confluence in the lower or middle or higher third of the EHBD.

The presence or absence of lithiasis in the gallbladder or EHBD was separately recorded.

Finally, the final diagnoses for malignancy (pancreatic or biliary cancers) and other diseases (intraductal papillary mucinous neoplasia (IPMN) and primary sclerosing cholangitis (PSC)) were also recorded.

### Statistical Analysis

Data are presented as counts and percentages for categorical variables and median and inter-quartile ranges (IQRs) for continuous variables. Categorical variables were compared using chi-squared or Fisher’s exact tests, as appropriate. Continuous variables were compared with the Kruskal–Wallis test. Inter-observer agreement correlation coefficient (ICC) with 95% confidence intervals (95% CI) was calculated for the evaluation of CDDP and EHBD lengths performed by the two radiologists; in the presence of a good correlation (ICC > 0.75), the average of the lengths of CDDP and EHBD assessed by the two radiologists was analysed for the purposes of the manuscript. The distribution of the ratio among CDDP and EHBD lengths was evaluated. Using the CDDP/EHBD ratio, the EHBDs were categorised in third parties according to the standard classification^4^: type 1, for CDDP/EHBD ratio of ≤ 33%; type 2, for CDDP/EHBD ratio of 34–66% and type 3, for CDDP/EHBD ratio of > 66%. In contrast, as per the percentile distribution of patients according to the aforementioned ratio (CDDP/EHBD) approximated every 5 units for simplicity in clinical use, the following three categories for classification of extrahepatic bile duct were subsequently identified: type 1 including subjects below the 25th percentile, type 2 including those between the 25th and 75th percentile, and type 3 including those above the 75th percentile. The area under ROC curve (AUROC) for each classification (standard vs new, according to Renzulli et al.) in identifying intra-pancreatic CD was calculated; AUROCs were compared using the DeLong test.

Differences between EHBD type and other bile duct anatomical variables or clinical symptoms were calculated. Univariate and multivariate logistic regression analyses were performed to assess the demographical and anatomical variables associated with a low implant of the CD in the EHBD according to the two abovementioned classifications and with clinical features (i.e. gallstones and choledochal lithiasis). The results are reported as odds ratio (OR) with 95% CI. An OR with an entire 95% CI of < 1 indicated that the covariate reduced the risks of finding the low implant of the CD in the EHBD and the risks of clinical features. In contrast, when the OR with an entire 95% CI was > 1, the covariate increased the abovementioned risks. An OR with 95% CI of 1 indicated that the covariate did not significantly influence these risks. The results obtained for the clinical endpoints from the multivariate analysis in the presence of two or more covariates influencing the risk were translated in a graphic form, suing special nomograms for logistic regression. Two-sided probability values have been reported; *P* values of < 0.05 were considered statistically significant. All the statistical analyses were performed using SPSS 13 (SPSS Inc., Chicago, IL, USA).

## Results

The study population comprised 1004 subjects who underwent MRCP during the study period, among which 543 subjects (54.1%) were women. The median age of the enrolled subjects was 63 years (IQR 51–73 years). The demographical, anatomical and clinical characteristics of the enrolled subjects are detailed in Table 1.

**Table 1:** Demographic characteristics, clinical symptoms, and anatomical variants of the study subjects

	Patients (*N* = 1004) *N* (%) or median (IQR)
Gender (F)	543 (54.1)
Age (years)	63 (51–73)
Indications to MRCP	
Choledochal lithiasis	79 (7.9)
Gallstones and/or Cholecystitis	153 (15.2)
Primary Sclerosing Cholangitis	168 (16.7)
Intraductal Papillary Mucinous Neoplasia	277 (27.6)
Increase in cholestasis enzymes	193 (19.2)
Pancreatic, biliary, and ampulla cancers	29 (2.9)
Evaluation before liver surgical resection	58 (5.8)
Main bile duct dilation in previous cholecystectomy	6 (6.0)
Biliary anatomical evaluation before surgery	27 (2.7)
Pancreatitis	14 (1.4)
Previous cholecystectomy	187 (18.6)
Intra-hepatic biliary duct variants according to^4^	
Type 1	635 (63.3)
Type 2	152 (15.1)
Type 3a	163 (16.2)
Type 3b	54 (5.4)
CDDP length (mm)	48 (39–57)
EHBD length (mm)	76 (68–85)
Ratio CDDP/EHBD (%)	64.4 (55.2–71.9)
CD insertion into the EHBD	
Lateral	764 (76.1)
Posterior	112 (11.2)
Medial	128 (12.7)
Intra-pancreatic CD	150 (14.9)
Lithiasis on MRCP	
Choledochal lithiasis	64 (6.4)
Gallstones	241 (24)

*CDDP*, cystic duct to duodenal papilla; *EHBD*, extrahepatic bile duct; *CD*, cystic duct; *MRCP*, magnetic resonance cholangiopancreatography

Overall, 37 subjects from the initial study population were excluded for the following reasons: 29 were excluded because of choledochal–jejunal anastomosis and 8 because of poor imaging quality.

The main indications to MRCP were as follows: follow-up of IPMN (277 patients, 27.6%), followed by the increase in cholestasis enzyme levels (193 patients, 19.2%), and follow-up for PSC (168 patients, 16.7%).

A previous cholecystectomy had been performed in 187 subjects (18.6%).

Most of the subjects showed normal anatomy of the intra-hepatic biliary tree (type 1 in 653 subjects, 63.3%), and the most common biliary variant was the type 3a (163 subjects, 16.2) according to the adopted classification.^3^ For CDDP and EHBD lengths which were assessed by two radiologists, the ICC was excellent (0.92, 95% CI 0.89–0.94 and 0.94, 95% CI 0.92–0.95) respectively.

The median ratio between the CDDP to the EHBD length was 64.4% (IQR 55.2%–71.9%). Most of the study subjects exhibited lateral CD insertion in the EHBD. A total of 150 subjects (14.9%) had intra-pancreatic CD.

### Classification of CD Implantation

**Fig. 1:**
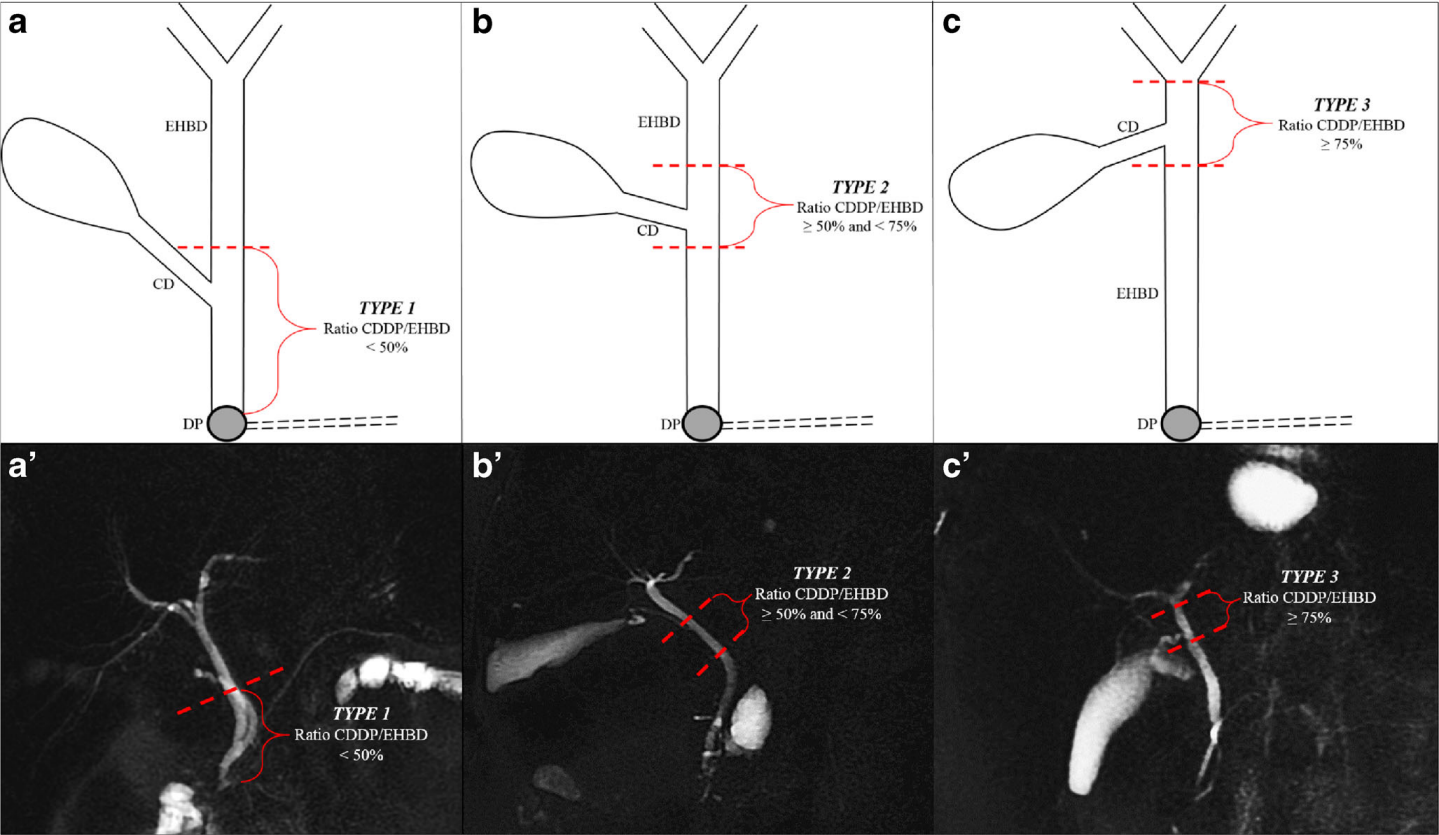
The new classification categorising the EHBD into three parts according to the percentile distribution of the CDDP/EHBD ratio. (**a**–**c**) Stylised scheme of the biliary tract. (A’–C’) MRCP images corresponding to each type. (A–A) Type 1 (below the 25th percentiles), CDDP/EHBD ratio ≤ 50%. (B–B’) Type 2 (between 25th and 75th percentile), CDDP/EHBD ratio > 50% and ≤ 75%. (C–C’) Type 3 (above the 75th percentiles), CDDP/EHBD ratio > 75%. *EHBD*, extrahepatic biliary ducts; *CD*, cystic duct; *DP*, duodenal papilla

Herein, a new classification for the EHBD was designed according to the percentile distribution of the CDDP/EHBD ratio, and the following categories were obtained: type 1 (below the 25th percentile) for CDDP/EHBD ratio of ≤ 50%, type 2 (between the 25th and 75th percentile) for CDDP/EHBD ratio of > 50% and ≤ 75% and type 3 (above the 75th percentile) for CDDP/EHBD ratio of > 75% (Fig. 1).

The demographic characteristics, clinical symptoms and anatomical variants of the patients according to the new and the standard classifications for EHBD were then compared, as detailed in Table 2. For both the classifications, significant differences were found among each category with respect to gender, whereas for median age, only those included in the new classification type 3 showed significantly lower age than others (*P* = 0.049). The CDDP/EHBD ratio used to obtain three categories for each classification was mainly influenced by the CDDP length (*P* < 0.001) and not by the EHBD length.

**Table 2:** Demographic characteristics, clinical symptoms, and anatomical variants of the study subjects according to the new and standard classifications for extrahepatic bile duct

	New classification for EHBD				Standard classification for EHBD			
	Type 1 (ratio CDDP/EHBD ≤ 50%)	Type 2 (ratio CDDP/EHBD > 50% and ≤ 75%)	Type 3 (ratio CDDP/EHBD > 75%)	*P*	Type 1 (ratio CDDP/EHBD ≤ 33%)	Type 2 (ratio CDDP/EHBD > 33% and ≤ 66%)	Type 3 (ratio CDDP/EHBD > 66%)	*P*
*N* of patients	181	675	148	-	36	549	423	-
Gender (M)	87 (48)	322 (47.7)	52 (35)	0.017	14 (38.9)	270 (49.5)	177 (41.8)	0.040
Age (years)	64 (52–75)	64 (51–72)	59.5 (49–71)	0.049	62.5 (51–75.5)	64 (52–73)	61 (50–72)	0.301
Previous cholecystectomy	37 (20.4)	130 (19.3)	20 (13.5)	0.210	5 (13.9)	115 (21.1)	67 (15.8)	0.086
Intra-hepatic biliary variants^4^				0.126				0.065
Type 1	98 (54.1)	438 (64.9)	99 (66.9)		16 (44.4)	335 (61.4)	284 (67.1)	
Type 2	35 (19.3)	99 (14.7)	18 (12.2)		7 (19.5)	92 (16.9)	53 (12.5)	
Type 3a	39 (21.6)	101 (15)	23 (15.5)		10 (27.8)	92 (16.9)	61 (14.4)	
Type 3b	9 (5)	37 (5.5)	8 (5.4)		3 (8.3)	26 (4.8)	25 (5.9)	
CDDP length (mm)	31 (25–36)	49 (43–56)	62 (53.5–70)	< 0.001	21 (16–25)	43 (36–49)	57 (50–64)	< 0.001
EHBD length (mm)	76 (66–85)	77 (68–85)	75 (67–86)	0.467	75 (64.5–83)	76 (68–85)	77 (69–86)	0.270
CD radial insertion in the EHBD				< 0.001				< 0.001
Lateral	66 (36.5)	559 (82.8)	139 (93.2)		3 (8.3)	377 (69.2)	384 (90.9)	
Posterior	21 (11.6)	86 (12.7)	5 (3.4)		3 (8.3)	81 (14.9)	28 (6.6)	
Medial	94 (51.9)	30 (4.4)	4 (2.7)		30 (83.4)	87 (15.9)	11 (2.5)	
Intra-pancreatic CD	117 (64.6)	32 (4.7)	1 (0.7)	< 0.001	33 (91.7)	111 (20.4)	6 (1.4)	< 0.001
MRCP findings								
Choledochal lithiasis	19 (19)	36 (10.8)	8 (12.5)	0.097	3 (16.7)	39 (13.9)	21 (10.6)	0.480
Gallstones	35 (35)	131 (39.3)	23 (35.9)	0.687	7 (38.9)	102 (36.4)	80 (40.2)	0.702
IPMN	40 (42.1)	192 (50.4)	45 (54.2)	0.230	10 (52.6)	145 (46.9)	122 (52.8)	0.385
PSC	23 (29.5)	115 (37.8)	30 (44.1)	0.181	5 (35.7)	84 (33.9)	79 (42)	0.217
Pancreatic a/o biliary cancers	18 (24.7)	35 (15.6)	9 (19.2)	0.214	3 (25)	36 (18)	23 (17.4)	0.808

*CDDP*, cystic duct to duodenal papilla; *EHBD*, extrahepatic bile duct; *CD*, cystic duct; *MRCP*, magnetic resonance cholangiopancreatography; *IPMN*, intraductal papillary mucinous neoplasia; *PSC*, primary sclerosing cholangitis; *a/o*, and/or

**Fig. 2:**
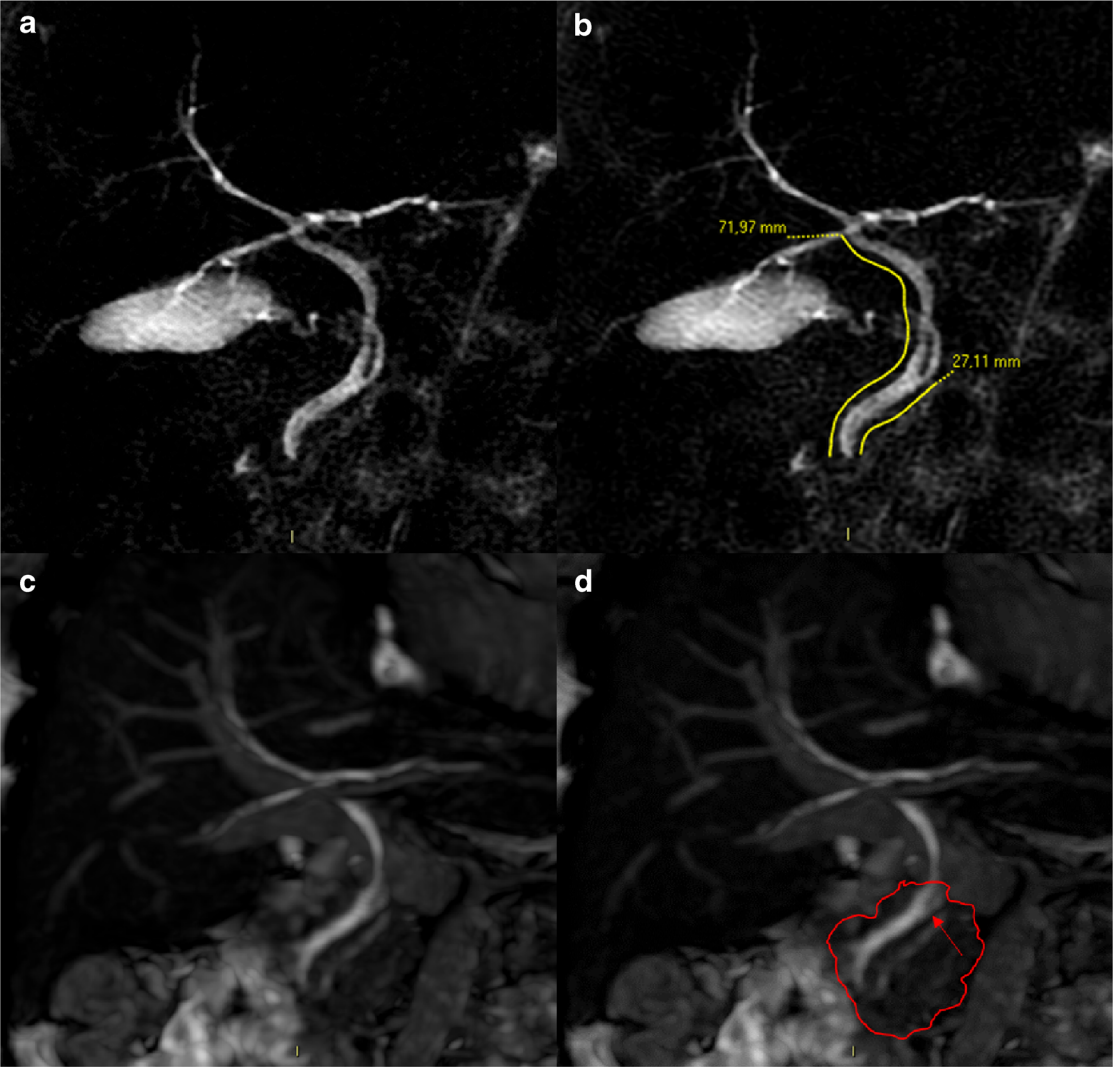
MRCP images of the same patient showing CDDP and EHBD assessment with quantitative method and anomalous CD insertions into EHBD. (**a**) MRCP image showing medial radial insertion of the CD into the EHBD. (**b**) CDDP and EHBD lengths measured on MRCP image, revealing a CDDP/EHBD ratio of 38%, corresponding to type 1 according to the new classification and to type 2 as per the standard classification. (**c**) Fiesta sequence showing the parenchyma of the pancreatic head that embraces the EHBD including the CD insertion as well perceptible in the panel (**d**), wherein the pancreatic head margins were traced with a red line. This case highlighted that the use of only the new EHBD classification for the diagnosis of type 1 allows the simultaneous diagnosis of low, intra-pancreatic CD with medial insertion

**Fig. 3:**
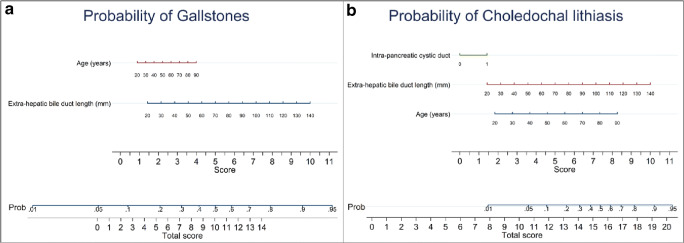
Nomograms. (**a**) Nomogram reporting a probability score for the gallstones expressed by the addition of single score for age and EHBD length. Older age was associated with a score of 1–4, while the EHBD length varied from 1.5–10. (**b**) Nomogram reporting a probability score for choledochal lithiasis; the influencing variables were intra-pancreatic CD, EHBD length, and age, composing a total score of 20

The radial implantation of the CD in the EHBD was lateral in most patients with Type 2 and Type 3 categories according to both the classifications, whereas it was medial in type 1 of as per the new classification (*P* < 0.001). Both the classifications were able to correctly associate the presence of an intra-pancreatic CD included in the type 1 category. In particular, the new and the standard classifications were able to identify 117 and 33 subjects with an intra-pancreatic CD from among 181 and 36 individuals in the type 1 categories, respectively. However, the standard classification did not identify all patients with intra-pancreatic CD using the type 1 category (33/150 cases, 22%) compared with the type 1 category of the new classification that allowed the detection of 78% of those with intra-pancreatic CD (117/150, *P* < 0.001). The AUROC in identifying patients with intra-pancreatic CD associated with the type 1 category of the standard classification versus the type 2 and type 3 categories was 0.608 (95% CI 0.575–0.642), whereas the AUROC associated with type 1 category of the new classification was 0.853 (95% CI 0.818–0.887), representing a significant difference (*P* < 0.001) (Figs. 2 and 3).

The multivariate analyses that aimed to evaluate the anatomical factors associated with type 1 EHBD of each classification confirmed that in both cases, the CDDP length and the medial radial insertion of the CD into the EHBD characterised this anatomical type (Table 3). However, the presence of an intra-pancreatic CD was a predictive factor only for type 1 EHBD according to the new classification (*P* < 0.001) (Table 3). In contrast, the multivariate analysis for the evaluation of factors associated with the presence of an intra-pancreatic CD confirmed that the CDDP/EHBD ratio (from which the extrahepatic bile duct classifications are designed) and the medial CD implantation in the EHBD were predictors of this anatomical variant (Table 4).

**Table 3:** Univariate and multivariate logistic regression analyses for the assessment of anatomical factors associated with extrahepatic bile duct type 1 according to Renzulli and type 1 as per the standard classification

	New classification according to Renzulli et al. for EHBD type 1				Standard classification for EHBD type 1			
	Univariate logistic regression (OR 95% CI)	*P*	Multivariate logistic regression (OR 95% CI)	*P*	Univariate logistic regression (OR 95% CI)	*P*	Multivariate logistic regression (OR 95% CI)	*P*
Gender (M)	1.111 (0.805–1.534)	0.522			0.742 (0.375–1.467)	0.390		
Age (years)*	1.009 (0.999–1.019)	0.086			1.002 (0.981–1.022)	0.890		
Intra-hepatic biliary variants^4^								
Type 1	Referent	-			Referent	-		
Type 2	1.639 (1.061–2.532)	0.026			1.868 (0.755–4.623)	0.177		
Type 3a	1.723 (1.133–2.62)	0.011			2.526 (1.125–5.682)	0.025		
Type 3b	1.095 (0.519–2.314)	0.810			2.276 (0.642–8.069)	0.203		
CDDP length (mm)*	0.757 (0.726–0.790)	< 0.001	0.794 (0.760–0.829)	< 0.001	0.648 (0.569–0.739)	< 0.001	0.668 (0.582–0.764)	< 0.001
EHBD length (mm)*	0.993 (0.981–1.006)	0.308			0.983 (0.957–1.010)	0.225		
CD radial insertion in the EHBD								
Lateral	Referent	-	Referent	-	Referent	-	Referent	-
Posterior	2.441 (1.426–4.177)	0.001	1.089 (0.512–2.318)	0.825	6.993 (1.394–35.086)	0.018	3.999 (0.442–36.186)	0.217
Medial	29.239 (18.340–46.615)	< 0.001	5.098 (2.276–11.415)	< 0.001	77.798 (23.304–259.726)	< 0.001	6.227 (1.235–31.388)	0.027
Intra-pancreatic CD	43.764 (27.552–69.516)	< 0.001	7.838 (3.885–15.814)	< 0.001	80.009 (24.156–265.001)	< 0.001		

*CDDP*, cystic duct to duodenal papilla; *EHBD*, extrahepatic bile duct; *CD*, cystic duct; *MRCP*, magnetic resonance cholangiopancreatography

*Per unit increase

**Table 4:** Univariate and multivariate logistic regression analyses for the assessment of the anatomical factors associated with the intra-pancreatic cystic duct

	Univariate logistic regression (OR 95% CI)	*P*	Multivariate logistic regression (OR 95% CI)	*P*
Gender (M)	0.913 (0.644–1.295)	0.610		
Age (years)*	1.013 (1.001–1.024)	0.031		
Intra-hepatic biliary variants^4^				
Type 1	Referent	-		
Type 2	1.932 (1.224–3.052)	0.005		
Type 3a	1.702 (1.077–2.691)	0.023		
Type 3b	1.647 (0.796–3.407)	0.178		
CDDP length (mm)*	0.855 (0.835–0.876)	< 0.001		
EHBD length (mm)*	0.996 (0.982–1.010)	0.554		
Ratio CDDP/EHBD (%)*	1.61^e−08^ (1.19^e−09^–2.17^e−07^)	< 0.001	3.48^−07^ (2.26^−08^–5.36^−06^)	< 0.001
New classification for EHBD				
Type 1 (ratio CDDP/EHBD ≤ 50%)	268.734 (36.732–1966.103)	< 0.001		
Type 2 (ratio CDDP/EHBD > 50% and ≤ 75%)	7.316 (0.992–53.970)	0.051		
Type 3 (ratio CDDP/EHBD > 75%)	Referent	-		
Standard classification for EHBD				
Type 1 (ratio CDDP/EHBD ≤ 33%)	764.5 (182.859–3196.235)	< 0.001		
Type 2 (ratio CDDP/EHBD > 33% and ≤ 66%)	17.775 (7.732–40.863)	< 0.001		
Type 3 (ratio CDDP/EHBD > 66%)	Referent	-		
CD radial insertion in the EHBD				
Lateral	Referent	-	Referent	-
Posterior	2.003 (1.047–3.834)	0.036	0.965 (0.447–2.084)	0.927
Medial	36.131 (22.347–58.419)	< 0.001	5.528 (2.939–10.395)	< 0.001
Previous cholecystectomy	1.056 (0.680–1.640)	0.809		

*CDDP*, cystic duct to duodenal papilla; *EHBD*, extrahepatic bile duct; *CD*, cystic duct

*Per unit increase

### Association Between the Anatomical Variants and Clinical Presentation

Among the 1004 patients, 282 patients (28.1%) underwent MRCP for the suspicion of lithiasis disease but showed no stones or biliary diseases; therefore, these patients were included as the control group. Subsequently, for each of the main indications for MRCP, multiple logistic univariate and multivariate analyses were performed to highlight the demographic and anatomical factors associated with each clinical pattern in comparison to that in the control group. Gallstones and choledochal lithiasis diagnosis at multivariate analysis were more likely to be present in older patients and those with longer EHBD (Tables 5, 6). The presence of an intra-pancreatic CD enabled the prediction of choledochal lithiasis (Table 6). The same results were obtained when gallstones and choledochal lithiasis were pooled because those pathological aspects mirror the same underlying disease (Supplementary Table 1).

**Table 5:** Logistic regression for the evaluation of demographical and anatomical variables associated with gallstones

	Univariate logistic regression (OR 95% CI)	*P*	Multivariate logistic regression (OR 95% CI)	*P*
Gender (M)	1.483 (1.031–2.134)	0.034		
Age (years)*	1.017 (1.005–1.029)	0.004	1.013 (1.001–1.025)	0.029
Intra-hepatic biliary variants^4^				
Type 1	Referent	-		
Type 2	1.489 (0.914–2.425)	0.110		
Type 3a	1.450 (0.888–2.368)	0.138		
Type 3b	1.493 (0.713–3.127)	0.288		
CDDP length (mm)*	1.018 (1.004–1.031)	0.011		
EHBD length (mm)*	1.025 (1.011–1.039)	0.001	1.021 (1.007–1.036)	0.004
Ratio CDDP/EHBD (%)*	1.649 (0.419–6.499)	0.474		
New classification for EHBD				
Type 1 (ratio CDDP/EHBD ≤ 50%)	0.960 (0.498–1.849)	0.903		
Type 2 (ratio CDDP/EHBD > 50% and ≤ 75%)	1.156 (0.663–2.016)	0.609		
Type 3 (ratio CDDP/EHBD > 75%)	Referent	-		
Standard classification for EHBD				
Type 1 (ratio CDDP/EHBD ≤ 33%)	0 .947 (0.352–2.545)	0.913		
Type 2 (ratio CDDP/EHBD > 33% and ≤ 66%)	0.852 (0.587–1.238)	0.402		
Type 3 (ratio CDDP/EHBD > 66%)	Referent	-		
CD radial insertion in the EHBD				
Lateral	Referent	-		
Posterior	1.492 (0.857–2.598)	0.157		
Medial	1.270 (0.748–2.157)	0.377		
Intra-pancreatic CD	0.919 (0.555–1.522)	0.743		

*CDDP*, cystic duct to duodenal papilla; *EHBD*, extrahepatic bile duct; *CD*, cystic duct; *MRCP*, magnetic resonance cholangiopancreatography

*Per unit increase

**Table 6:** Logistic regression for the evaluation of demographical and anatomical variables associated with choledochal lithiasis

	Univariate logistic regression (OR 95% CI)	*P*	Multivariate logistic regression (OR 95% CI)	*P*
Gender (M)	1.322 (0.778–2.246)	0.302		
Age (years)*	1.063 (1.041–1.086)	< 0.001	1.057 (1.034–1.081)	< 0.001
Intra-hepatic biliary variants^4^				
Type 1	Referent	-		
Type 2	1.655 (0.866–3.161)	0.127		
Type 3a	0.966 (0.457–2.041)	0.928		
Type 3b	0.490 (0.112–2.138)	0.342		
CDDP length (mm)*	1.016 (0.997–1.036)	0.103		
EHBD length (mm)*	1.053 (1.032–1.075)	< 0.001	1.044 (1.022–1.066)	< 0.001
Ratio CDDP/EHBD (%)*	0.152 (0.023–1.017)	0.052		
New classification for EHBD				
Type 1 (ratio CDDP/EHBD ≤ 50%)	1.642 (0.672–4.013)	0.277		
Type 2 (ratio CDDP/EHBD > 50% and ≤ 75%)	0.848 (0.375–1.922)	0.694		
Type 3 (ratio CDDP/EHBD > 75%)	Referent	-		
Standard classification for EHBD				
Type 1 (ratio CDDP/EHBD ≤ 33%)	1.695 (0.453–6.343)	0.433		
Type 2 (ratio CDDP/EHBD > 33% and ≤ 66%)	1.372 (0.780–2.413)	0.273		
Type 3 (ratio CDDP/EHBD > 66%)	Referent	-		
CD radial insertion in the EHBD				
Lateral	Referent	-		
Posterior	1.263 (0.560–2.846)	0.574		
Medial	2.126 (1.085–4.165)	0.028		
Intra-pancreatic CD	2.542 (1.379–4.687)	0.003	2.354 (1.204–4.605)	0.012
Previous cholecystectomy	1.508 (0.833–2.729)	0.175		

*CDDP*, duct to duodenal papilla; *EHBD*, extrahepatic bile duct; *CD*, cystic duct; *MRCP*, magnetic resonance cholangiopancreatography

*Per unit increase

## Discussion

Biliary tree diseases are extremely widespread worldwide and affect a large part of the adult population.^11^ Precise evaluation of the biliary tract anatomy, including CD, is essential for surgeons to ensure effective and safe interventions, thereby reducing intra-operative and post-operative complications.^4^,^9^,^20^ Moreover, variations of biliary tract anatomy, including CD, are associated with biliary tract pathologies.^4^,^8^,^20^ Therefore, accurate knowledge of the biliary anatomy and its variations is also required for radiologists.

The present study population comprised 1004 consecutive subjects who had undergone MRCP for various clinical indications over a period of 29 months. Previous studies with similar aims involved different study populations. Gupta et al. enrolled a total of 500 patients in 24 months, and Tsitouridis et al. enrolled 863 individuals in 60 months.^9^,^14^ Therefore, our study population is more representative of the target population due to its large size obtained in a short period.

The subjects enrolled in this study had a median age of 63 years. This finding differs from that reported by other series^9^ and is not comparable with findings of other studies wherein the age of patient populations was not considered.^14^,^21^ The median age of patients in the study by Gupta et al.^9^ was smaller (42.3 years) than that of patients in the present series, probably due to different indications for MRCP. In fact, in the study by Gupta et al.,^9^ the oncological indications for MRCP were not reported and were probably (but un-specified) included by the authors in the ‘Miscellaneous’ item, accounting for 17.4% of the total cases. However, our institution is a tertiary centre for oncological pathologies, such as hepato–bilio–pancreatic neoplasms and is well-known for oncological diseases, such as IPMN, accounting for more than one-fourth of all our indications for MRCP and are usually correlated with older age.^22^,^23^ Therefore, the older age of patients in our study population resulted in more oncological indications. However, these data can be considered even more significant in the terms of increased longevity.

In the present study, a normal intra-hepatic biliary tree anatomy was reported in 63.3% of the patients. This result is in agreement to the findings reported by Gupta et al. and Cucchetti et al. (65.7% and 64.5%, respectively).^4^,^9^ The most frequent atypical intra-hepatic biliary tree patterns of our series were type 3a (16.2%) and type 2 (15.1%), analogous to that reported in other series.^9^,^24^,^25^ However, these values were not in line with those reported by Cucchetti et al., with a slight prevalence of type 2 (14%) variant over type 3a (12%).^4^ One possible explanation for this difference is the different characteristics of the study population. In particular, in the series of Cucchetti et al.,^4^ only patients who underwent whole liver transplantation were enrolled, and a number of patients corresponding to half of the final population were excluded from the final analysis due to unavailable or unsatisfactory imaging of the intra-hepatic biliary anatomy. In contrast, our study employed a large sample that comprised consecutive patients and non-selected for clinical indications. However, the findings of the abovementioned studies remain congruous with our findings because the overall number of ‘typical’ anatomical patterns of intra-hepatic biliary tree represents approximately 2/3 of all findings, and the other anatomical variations cover the remaining one-third.

The anatomical features of CD and EHBD in our study population were evaluated with a quantitative method obtained by precisely measuring the CDDP and EHBD lengths that allow the calculation of their ratio. The median ratio between CDDP and EHBD was 64.4%. To the best of our knowledge, this assessment has not been performed previously and represents the innovative characteristics of our study compared with previous studies that have always reported the EHBD anatomy only using a descriptive approach.^1^–^3^,^12^ The CDDP/EHBD ratio was significantly influenced by the CDDP length (*P* < 0.001) and not by the EHBD length. The possible explanation could be that the CDDP length depends on the level at which the CD joins EHBD that usually varies widely. Conversely, the EHBD length is influenced by the body mass and height of patients^1^ that did not widely differ among the European subjects in our large study population.

The radial CD insertion into the EHBD resulted to be lateral in 76.1%, according to the vast majority of published series.^1^,^3^,^9^,^10^,^14^ Our data differ from that reported by Tsitouridis et al. wherein the lateral insertion of CD accounted for 31.8%.^14^ However, this difference was only apparent because the authors enrolled only patients with suspected CBD stones. In fact, in our series, the low and intra-pancreatic CD insertion correlates with a higher probability of lithiasis, and most CD with a low implant had medial insertion, thus explaining the differences with Tsitouridis experience.

Approximately 15% of our patients demonstrated an intra-pancreatic CD insertion into the EHBD. This feature represents another uniqueness of our study, given that none of the similar previous series focused on this evaluation. The few authors describing this pattern have defined intra-pancreatic CD as an extremely rare and almost negligible variant of the low CD insertion.^1^,^3^

In our study, the EHBDs were divided into three main thirds using the ‘old’ standard classification to perform proper comparison between our data and those reported by previously published series. However, our assessment of the EHBD was performed using a quantitative method, which is different from that adopted in other series.^1^–^3^,^9^,^10^,^14^ Moreover, knowing the clinical significance of a correct evaluation of CD insertion and the utility to identify at the same time low and intra-pancreatic CD,^1^,^10^,^14^ a new classification for the EHBD according to percentile distribution of the CDDP/EHBD ratio was designed, obtaining the following categories: type 1 for CDDP/EHBD ratio of ≤ 50%, type 2 for CDDP/EHBD ratio of > 50% and ≤ 75% and type 3 for CDDP/EHBD ratio of > 75%.

Applying the ‘old’ classification to our study population, only 22% of the intra-pancreatic CD would have been classified as type 1 category (insertion in the lower third of the EHBD). However, using the new classification, 78% of intra-pancreatic CDs were included in the type 1 category, with a statistically significant difference. A possible explanation is that the parenchyma of the pancreatic head embraces the EHBD more than its lower third up to its middle third. Therefore, most intra-pancreatic CD presenting a middle third insertion will remain unidentified using the type 1 category of the standard classification. However, type 1 of the proposed classification involving a wide portion of EHBD (up to 50%) is able to identify the vast majority of intra-pancreatic CD.

In addition to the CDDP/EHBD ratio, another anatomical factor that significantly correlates with the EHBD type 1 category of both the classifications is the medial implantation of the CD, although with more robust significance in our new classification. In a previous series that used the standard classification, a CD insertion in the ‘lower’ third of EHBD had no impact on medial insertion.^10^,^14^

This new classification could be very useful in clinical practice because it allows radiologists to more easily define the central point (the midpoint) of the EHBD rather than measuring its third portion. Therefore, if the CD joins the EHBD in its lower half (type 1), there will be a high probability of intra-pancreatic CD diagnosis with medial insertion. To our knowledge, the present study is the first in the literature to demonstrate that intra-pancreatic CD is strongly associated with choledochal lithiasis. This could be attributable to the compressive effect of the pancreatic parenchyma on the EHBD that could cause bile stasis and consequent stone formation. Therefore, the use of our new classification is advocated owing to its ability to predict choledochal lithiasis and identifying the majority of intra-pancreatic CD by using the EHBD type 1 category.

Our results confirmed that the demographic and anatomical variables associated with gallstones and choledochal lithiasis are age and EHBD length. This association was already established because (a) the number of patients affected by lithiasis significantly increases with age and (b) a long EHBD probably represents a major risk factor for stone diseases, such as bile stasis and bacterial overlap facilitate stone formation.^11^,^13^ The same results were obtained when patients with gallstones and choledochal lithiasis (lithiasic disease in general) were pooled.

Another novelty of this study is the introduction of nomograms for risk estimation of gallstones and choledochal lithiasis by the radiologist on bases of the demographic and MRCP data. The nomogram is a two-dimensional diagram that allows the approximate graphical calculation of a function, such as the multivariate analysis. These graphical instruments are easy-to-use and enable rapid calculation of the pathological risks for a single patient in the era of tailored medicine.

This study has certain limitations. First, the study population comprised non-selected consecutive patients referred to a highly specialised centre, and the control group did not comprise healthy volunteer patients. However, we considered the MRCP group with negative findings as the control group. Second, the population sample was investigated with MRCP without comparisons to the results of other endoscopic imaging modalities, such as endoscopic ultrasound or endoscopic retrograde cholangiopancreatography. However, it is widely established that MRCP represents the most accurate imaging modality for assessment of the entire biliary tree.^15^–^18^ The third possible limitation could be that the new EHBD classification has been arbitrarily constructed based on percentile distribution. However, the category delineations were driven by the following four key points: the use of a quantitative classification that increases the reliability of the method, the construction of a ‘common-finding’ category that includes most patients between the 25th and 75th percentiles, the clinical meaning of type 1 that should have included most cases of intra-pancreatic CD that are related to biliary tree disease, and finally, the easy-to-use MRCP modality and classification that enables the radiologist to perform an initial evaluation for at-risk patients by splitting the EHBD first in half and then in two other parts in the upper level.

In conclusion, the new methodological approach used for assessing the biliary tree anatomy, the large number of patients involved, and the considerable anatomic-pathological correlations are the strengths of this study and make it an important research on this subject in Europe. The new classification of CD implantation enables the identification of most low intra-pancreatic CD, correlated to a high risk of choledochal lithiasis, in a single category (type 1), facilitating an easy detection on imaging. Further studies are needed to validate this classification in order to translate its use in clinical practice. The nomogram we used could be an easy-to-use tool for estimating the risk of gallstones and choledochal lithiasis diagnosis made by the radiologist according to the sole demographics and MRCP data.

## Supplementary Information

ESM 1 (DOCX 47 kb)
