# Supplementary material for: A New Quantitative Classification of the Extrahepatic Biliary Tract Related to Cystic Duct Implantation
Source: J Gastrointest Surg. 2020 Dec 2;25(9):2268–79. doi: 10.1007/s11605-020-04852-8 (PMC8484130; doi:10.1007/s11605-020-04852-8)
Supplement: Supplementary file 1 — (DOCX 47 kb) [file 11605_2020_4852_MOESM1_ESM.docx]

**Supplementary Table 1.** Logistic regression analyses for the evaluation of demographical and anatomical variables associated with choledochal and gallbladder lithiasis.

|  | **Univariate logistic regression**  **(OR 95% CI)** | **P** | **Multivariate logistic regression**  **(OR 95% CI)** | **p** |
| --- | --- | --- | --- | --- |
| **Gender (M)** | 1.392 (0.975-1.987) | 0.069 |  |  |
| **Age (years)*** | 1.026 (1.014-1.038) | <0.001 | 1.022 (1.010-1.034) | <0.001 |
| **Intra-hepatic biliary variants [4]** |  |  |  |  |
| Type 1 | Referent | - |  |  |
| Type 2 | 1.626 (1.005-2.631) | 0.048 |  |  |
| Type 3a | 1.319 (0.813-2.141) | 0.262 |  |  |
| Type 3b | 1.181 (0.565-2.465) | 0.660 |  |  |
| **CDDP length (mm)*** | 1.018 (1.004-1.031) | 0.010 |  |  |
| **EHBD length (mm)*** | 1.032 (1.017-1.046) | <0.001 | 1.027 (1.012-1.041) | <0.001 |
| **Ratio CDDP/EHBD (%)*** | 1.013 (0.266-3.847) | 0.985 |  |  |
| ***New classification for EHBD*** |  |  |  |  |
| Type 1  (Ratio CDDP/EHBD ≤50%) | 1.196 (0.633-2.258) | 0.581 |  |  |
| Type 2  (Ratio CDDP/EHBD >50% and ≤75%) | 1.114 (0.646-1.918) | 0.698 |  |  |
| Type 3  (Ratio CDDP/EHBD >75%) | Referent | - |  |  |
| ***Standard classification for* EHBD** |  |  |  |  |
| Type 1  (Ratio CDDP/EHBD ≤33%) | 1.211 (0.461-3.180) | 0.697 |  |  |
| Type 2  (Ratio CDDP/EHBD >33% and ≤66%) | 0.857 (0.594-1.236) | 0.408 |  |  |
| Type 3  (Ratio CDDP/EHBD >66%) | Referent | - |  |  |
| **CD insertion in the EHBD** |  |  |  |  |
| Lateral | Referent | - |  |  |
| Posterior | 1.613 (0.929- 2.800) | 0.089 |  |  |
| Medial | 1.414 (0.839-2.383) | 0.193 |  |  |
| **Intra-pancreatic CD** | 1.258 (0.773-2.047) | 0.356 |  |  |

*CDDP*: Cystic duct to duodenal papilla; *EHBD*: Extra-hepatic bile duct; *CD*: Cystic duct; *MRCP*: Magnetic Resonance Cholangiopancreatography.

*****per unit increase
